# Supplementary material for: WordCommentsAnalyzer: A windows software tool for qualitative research
Source: F1000Res. 2018 Sep 4;7:536. Originally published 2018 May 3. [Version 2] doi: 10.12688/f1000research.14819.2 (PMC6137411; doi:10.12688/f1000research.14819.2)
Supplement: Supplementary file 3 [file f1000research-7-17614-s0002.tgz › b2a79496-4ada-47c4-9682-a99ce867836d.zip!/Eight Iranian Health Journals/Salmand_Iranian Journal of Ageing _7.docx]

© Salmand_Iranian Journal of Ageing . This work is licensed under CC BY-NC 4.0 (https://creativecommons.org/licenses/by-nc/4.0/).

Volume & Issue: دوره 11 - شماره 3

Title: وضعیت تغذیه و برخی عوامل مرتبط با آن در سالمندان دیابتی شهر کرمان ، 1394

http://salmandj.uswr.ac.ir/article-1-1086-fa.html

اهداف سوء‌تغذیه اختلالی بالینی و عاملی خطرزا در سالمندان دیابتی محسوب می‌شود که ممکن است وضعیت سلامتی آن‌ها را وخیم‌تر کند. این مطالعه باهدف بررسی وضعیت تغذیه سالمندان دیابتی در شهر کرمان انجام شد.  مواد و روش ها مطالعه حاضر مطالعه‌ای مقطعی بود که در آن دویست نفر از سالمندان دیابتی بررسی شدند. برای جمع‌آوری اطلاعات درباره وضعیت تغذیه از پرسش‌نامه مختصر ارزیابی وضعیت تغذیه‌ای MNA و برای تحلیل داده‌ها از شاخص‌های توصیفی و نیز برای بررسی ارتباط متغیرها از آزمون‌های آماری خی دو، آزمون تی، تحلیل واریانس یک‌طرفه و همبستگی استفاده شد. یافته ها 5/3درصد سالمندان دیابتی بررسی‌شده به سوء‌تغذیه مبتلا بودند و تقریباً نیمی از آن‌ها (51/6درصد) وضعیت تغذیه‌ای طبیعی داشتند. رابطه بین جنسیت و وضعیت تغذیه معنا‌دار بود؛ به‌طوری‌که زنان سالمند بیشتر از مردان دچار سوء‌تغذیه بودند (036/0=P). همچنین بین مدت‌زمان ابتلا به دیابت و وضعیت تغذیه ارتباط منفی و معناداری وجود داشت (008/0=P و 192/0-=r). نتیجه گیری باتوجه‌به یافته‌های این مطالعه، علاوه‌بر اهمیت حفظ و ارتقای وضع موجود در سالمندان دیابتی با وضعیت تغذیه طبیعی، باید به افراد درمعرض سوءتغذیه و افراد مبتلا به سوءتغذیه توجه ویژه‌ای شود.

Title: اثربخشی مشاوره گروهی با رویکرد معنادرمانی برکاهش احساس تنهایی مردان سالمند

http://salmandj.uswr.ac.ir/article-1-1102-fa.html

اهداف در جوامع امروزی سالمندان یکی از گروه‌های اجتماعی روبه‌رشد هستند که باتوجه‌به هرم سنی اهمیت روزافزونی یافته‌اند. پژوهش حاضر باهدف بررسی تأثیر مشاوره گروهی با رویکرد معنادرمانی به‌عنوان روش توان‌بخشی بر کاهش احساس تنهایی مردان سالمند انجام شده است.  مواد و روش ها این تحقیق تجربی با به‌کارگیری طرح نیمه‌آزمایشی از نوع پیش‌آزمون پس‌آزمون با گروه کنترل اجرا شده است. جامعه آماری شامل تمامی سالمندان مرد کانون جهان‌دیدگان شهر تهران بود. در این مطالعه از طریق نمونه‌گیریِ دردسترس 36 نفر انتخاب و به‌صورت تصادفی به دو گروه 18 نفری آزمایش و کنترل تقسیم شدند. گروه آزمایش به‌مدت هشت جلسه 90 دقیقه‌ای (هفته‌ای یک‌بار) معنادرمانی را آموزش دیدند. به‌فاصله یک ماه از اجرای پس‌آزمون، آزمون پیگیری گرفته و نمره پرسش‌نامه احساس تنهایی دهشیری و همکاران قبل و بعداز مداخله مقایسه شد.  یافته ها نتایج این پژوهش با استفاده از تحلیل کوواریانس نشان داد که مشاوره گروهی با رویکرد معنادرمانی بر کاهش احساس تنهایی مردان سالمند تأثیر معنا‌داری دارد (01/0>P). به‌علاوه در مرحله پیگیری کاهش معنا‌داری همچنان تداوم داشت.  نتیجه گیری نتایج این مطالعه حاکی‌از آن بود که مشاوره گروهی با رویکرد معنادرمانی به‌عنوان روش توان‌بخشی در بهبود احساس تنهایی سالمندان تأثیر بسزایی دارد؛ بنابراین می‌توان روش به‌کاررفته در پژوهش حاضر را برای مداخلات روانی در سالمندان مرد، روش مناسبی قلمداد کرد.

Title: الگوهای گذران وقت سالمندان ایرانی و هم‌بسته‌های اقتصادی‌اجتماعی آن

http://salmandj.uswr.ac.ir/article-1-1132-fa.html

اهداف الگوهای گذران وقت ازیک‌سو بر سلامت و کیفیت زندگی سالمندان و ازسوی‌دیگر بر تفاوت‏های بافتاری هر جامعه در الگوهای گذران وقت تأثیر می‌گذارد. براین‌اساس هدف مقاله حاضر بررسی الگوهای گذران وقت سالمندان ایرانی و عوامل اجتماعی و جمعیت‌شناختی مؤثر بر آن است. مواد و روش ها در این مطالعه الگوهای گذران وقت و عوامل اقتصادی و اجتماعی مؤثر بر آن با استفاده از تحلیل ثانویه داده‏های پیمایش گذران وقت ایرانی‌ها بررسی شد که در سال 1388 مرکز آمار ایران به‌شیوه نمونه‏گیری دومرحله‏ای طبقه‌بندی‌شده گردآوری کرده بود. جامعه آماری پژوهش حاضر را 3516 سالمند 60 ساله و بیشترِ ساکن نقاط شهری کشور تشکیل می‌دادند. داده‌های حاصل از این تحقیق با روش‌های آماری توصیفی‌تحلیلی نظیر تحلیل واریانس و طبقه‌بندی چندگانه تجزیه‌وتحلیل شد. یافته ها یافته‏های مطالعه نشان داد بیشترین میانگین گذران وقت سالمندان ایرانی به‌ترتیب نگهداری و مراقبت شخصی، فعالیت‌های فراغتی، کار و خدمات خانگی بدون‌دستمزد، فعالیت‏های مذهبی، فعالیت‌های اجتماعی داوطلبانه و درنهایت فعالیت‌های دستمزدی شغلی است. این الگوها در میان گروه‌های مختلف اجتماعی تفاوت معناداری داشت.  نتیجه گیری نتایج تحلیل چندمتغیره نشان داد سن، جنسیت، وضعیت فعالیت، وضعیت اقتصادی، سطح تحصیلات، ترتیبات زندگی و وضعیت معلولیت و ناتوانی مهم‌ترین و قوی‌ترین متغیرهای اثرگذار بر الگوهای گذران وقت سالمندان ایرانی محسوب می‌شود.

Title: بررسی دیدگاه و تجربیات دانشجویان از زندگی با پدربزرگ و مادربزرگ سالمند

http://salmandj.uswr.ac.ir/article-1-696-fa.html

اهداف رابطه نوه‌ها با پدربزرگ‌ها و مادربزرگ‌ها به دلایل مختلف بسیارمهم و درخورتوجه و حساس است. تبیین تجربه نوه‌ها از پدربزرگ و مادربزرگ می‌تواند مجسم‌کننده ارتباط این دو نسل و اساس برنامه تعامل خانوادگی باشد؛ ازاین‌رو هدف مطالعه حاضر تبیین دیدگاه‌ها و تجربه‌های دانشجویان از زندگی با پدربزرگ و مادربزرگ سالمند است. مواد و روش ها این مطالعه کیفی براساس نمونه‌گیری مبتنی‌بر هدف از پنجاه دانشجو برای شرکت در مصاحبه‌های نیمه‌ساختاریافته دعوت و از آنان خواسته شد دیدگاه‌ها و تجربه‌های مثبت و منفی خود را درباره پدربزرگ و مادربزرگ خود بازگو کنند. مصاحبه‌ها روی کاغذ نوشته و کدگذاری و براساس روش تحلیل محتوا تجزیه‌وتحلیل شد. یافته ها مضامین گرفته‌شده از این مطالعه دوگانگی را در احساس دانشجویان دربرابر پدربزرگ و مادربزرگ خود نشان می‌دهد. دو درون‌مایه اصلی «مهربانی و دلسوزی» و «رنجش و دلخوری» استخراج شد. زیرطبقات مهربانی و دلسوزی شامل معنویت و گذشت و محبت بی‌دریغ و زیرطبقات رنجش و دلخوری شامل تبعیض بین خانواده، دخالت در کارها، شِکوه و گلایه‌کردن و رک‌گویی بوده است. نتیجه گیری دانشجویان در تجربیات خود از سالمندان درس‌هایی آموخته بودند. والدین باید پل ارتباطی این دو نسل باشند؛ زیرا بودن و دیدن این رفتارها در تکوین و شکل‌دهی شخصیت این نسل تأثیرگذار است.

Title: ارتباط دعا با سلامت معنوی سالمندان ایرانی مبتلا به حوادث عروق مغزی (CVA)

http://salmandj.uswr.ac.ir/article-1-1210-fa.html

زمینه و هدف: سکته مغزی از اصلی ترین مشکلات سلامت سالمندان در دنیا می باشد. بیماری ممکن است بحران های معنوی زیادی در فرد ایجاد کند.هدف از این تحقیق، بررسی ارتباط دعا با سلامت معنوی در سالمندان مبتلا به حوادث عروق مغزی می باشد. روش بررسی: در این مطالعه همبستگی 142 بیمار مبتلا به حوادث عروق مغزی بخش نورولوژی بیمارستان بوعلی سینا ساری مورد بررسی قرار گرفتند. ابزار گرد آوری داده ها پرسشنامه ی استاندارد سلامت معنوی پلوتزین و الیسون و پرسشنامه ی دعا  مراویگلیا بود. . داده ها با استفاده از نرم افزار SPSS20 و با کمک شاخص های آمارتوصیفی( میانگین و انحراف معیار) و ضریب همبستگی پیرسون  و تی مستقل وتحلیل واریانس تجزیه و تحلیل شد. یافته ها: از 142 بیمار مورد بررسی 64نفر(45.1%) مرد و 78نفر(54.9%) زن بودند. میانگین سنی آنها 7.67±72.97سال با دامنه سنی 95-60سال بود. میانگین نمره ی کل دعا 25.50±181.62 و بالاترین میزان نمره ی دعا مربوط به تناوب دعا بامیانگین  22.3±96.47بوده است. میانگین نمره کل سلامت معنوی 18.89±101.28 نیز بالا بود . همبستگی بین دعا و سلامت معنوی مستقیم بوده و ارتباط معنی داریبین آنها وجود داشتp<0/001, r=0/602)). همچنین ارتباط معنی داری بین سن و سلامت معنوی وجود داشت(p<0/001, r=0/607).ارتباط معنی داری بین سلامت معنوی با تحصیلات، شغل و وضعیت تاهل وجود نداشت. نتیجه گیری: انجام فرائض مذهبی و دعا می تواند منبعی قوی برای تطابق با شرایط تنش زا و حمایت های روانی- اجتماعی در بیماران با سکته مغزی باشد .

Title: ارتباط سلامت معنوی و اضطراب در سالمندان بستری در بخش مراقبت‌های ویژه قلبی

http://salmandj.uswr.ac.ir/article-1-1114-fa.html

اهداف اضطراب و دیسترس‌های معنوی یکی از مشکلات شایع بیماران بستری در بخش‌های مراقبت ویژه قلبی است. این مشکل در سالمندان به‌دلیل اختلال در سازوکارهای سازگاری آنان بیشتر احساس می‌شود. مطالعات نشان داده سلامت معنوی با وضعیت جسمانی و روانی افراد ارتباط دارد؛ اما ارتباط سلامت معنوی با اضطراب شخصیتی و موقعیتی هنوز واضح نیست. ازاین‌رو پژوهش حاضر باهدف بررسی ارتباط بین سلامت معنوی با اضطراب در سالمندان بستری در بخش مراقبت‌های ویژه قلب انجام شد. مواد و روش ها پژوهش حاضر مطالعه‌ای توصیفی و از نوع همبستگی بود. 66 بیمار قلبی سالمند بستری در بخش مراقبت‌های ویژه قلب به روش نمونه‌گیری دردسترس انتخاب شدند. پرسش‌نامه سلامت معنوی الیسون و پالوتزین و اضطراب اسپیلبرگر برای بیماران تکمیل شد. داده‌ها با استفاده از نسخه 21 نرم‌افزار SPSS و آزمون‌های آماری توصیفی، ضریب همبستگی پیرسون، تی مستقل و آنووا در سطح معنا‌داری P<0/05 تجزیه‌وتحلیل شد. یافته ها بیشترین شرکت‌کنندگان (7/69درصد) در رده سنی 60 تا 70، زن (1/59درصد)، متأهل (6/63درصد) و بی‌سواد (6/57درصد) بودند. میانگین نمره سلامت معنوی 79/55 و اضطراب شخصیتی 21/9±18/45 در محدوده متوسط و میانگین نمره اضطراب موقعیتی 9/29±44/58 و در محدوده بالا بود. نتایج پژوهش ارتباط منفی معنا‌داری را بین سلامت معنوی وجودی و مذهبی با اضطراب موقعیتی و شخصیتی نشان داد (P<0/05). همچنین بیشترین رابطه همبستگی بین سلامت معنوی و اضطراب شخصیتی وجود داشت (P=0/003). نتیجه گیری باتوجه‌به ارتباط چشمگیر بین سلامت معنوی و اضطراب، پرستاران می‌توانند با شناسایی و رفع نیازهای معنوی بیماران سالمند اضطراب آنان را کاهش دهند.

Title: بررسی همراهی ژن (rs1695) GSTP1 با بیماری آلزایمر دیررس در جمعیت ایرانی و ارتباط این پلی‌مورفیسم در میان‌کنش با APOE-ԑ4

http://salmandj.uswr.ac.ir/article-1-1145-fa.html

اهداف گلوتاتیون S-ترانسفرازها آنزیم‌های دتوکسیفیه‌کننده‌ای است که گونه‌های اکسیژن فعال اضافی (ROS) را از سلول حذف می‌کند. شواهد نشان می‌دهد که در چند مرحله از بیماری‌های نورودجنراتیو مانند بیماری آلزایمر استرس اکسیداتیو نقش دارد. رادیکال‌های آزاد و مولکول‌های مشابه که به‌عنوان گونه‌های اکسیژن فعال (ROS) طبقه‌بندی شده است، توانایی تغییرات اکسیداتیو را در داخل سلول دارند. در این مطالعه ارتباط پلی‌مورفیسم ژن GSTP1 (rs1695) با خطر ابتلا به آلزایمر دیررس و میان‌کنش این پلی‌مورفیسم با ژن APOE بررسی شد. ژن APOE در حفاظت و ترمیم نورون‌ها درگیر است. مواد و روش ها مطالعه حاضر از نوع موردی‌شاهدی بود. در این پژوهش 280 فرد مبتلا به بیماری آلزایمر به‌عنوان حجم نمونه برای ژن GSTP1 و 168 نفر به‌عنوان گروه شاهد انتخاب شدند. برای بررسی اثر ژنوتیپ‌ها و آلل‌های حاصل از پلی‌مورفیسم rs1695 ژن GSTP1 با بیماری آلزایمر و ارتباط این پلی‌مورفیسم‌ها در میان‌کنش با APOE-ԑ4 از روش PCR-RFLP استفاده شد. نتایج به‌دست‌آمده با استفاده از نسخه شانزدهم نرم‌افزار SPSS و آزمون ناپارامتری خی-2 و رگرسیون لجستیک تجزیه‌وتحلیل شد.  یافته ها نتایج نشان می‌دهد فراوانی ژنوتیپ هتروزیگوت (AG) در جمعیت گروه کنترل درمقایسه‌با بیمار برای rs1695 ژن GSTP1 بیشتر بوده است [(0/38-0/84) 0/57=OR (P=0/005)]. در توزیع ژنوتیپی هتروزیگوت در بین افراد کنترل و بیمار در جمعیت زنان نیز تفاوت معنی‌داری مشاهده شد (P=0/02). همچنین اثر متقابل بین ژنوتیپ هتروزیگوت GSTP1 (rs1695) و فقدان آلل APOE-ԑ4 در افراد وجود داشت (P=0/001).  نتیجه گیری باتوجه‌به نتایج این مطالعه ژنوتیپ هتروزیگوت (rs1696) ژن GSTP1 در‌برابر AD به‌عنوان عامل محافظتی عمل می کند.

Title: تأثیر برنامه ذهن‌آگاهی بر فشار مراقبتی مراقبان سالمندان مبتلا به آلزایمر

http://salmandj.uswr.ac.ir/article-1-999-fa.html

اهداف آلزایمر بیماری دژنراتیو مغزی و مزمن و پیش‌رونده است که بر حافظه و شناخت و توانایی افراد در انجام فعالیت‌های روزانه تأثیر می‌گذارد و در بسیاری از موارد به مراقب همیشگی نیاز خواهد داشت. بیماران مبتلا به آلزایمر دچار کاهش ظرفیت حافظه فعال می‌شوند. مراقبت از فرد مبتلا به آلزایمر می‌تواند هزینه‌های جسمی و عاطفی و مالی فراوانی داشته باشد. یکی از مشکلاتی که در مراقبت از سالمندان مبتلا به آلزایمر وجود دارد، مسئله فشار مراقبتی است. فشار مراقبتی بر سلامت مراقبان تأثیرگذار خواهد بود؛ به‌طوری‌که بسیاری از آنان استرس زیادی را تجربه می‌کنند. روش‌های مختلفی برای کاهش فشار ناشی‌از مراقبت در مراقبان همچون تکنیک‌های آرام‌سازی معرفی شده است. هدف این مطالعه تعیین تأثیر برنامه ذهن‌آگاهی بر فشار مراقبتی مراقبان سالمندان مبتلا به آلزایمر مراجعه‌کننده به انجمن آلزایمر ایران بود. مواد و روش ها این پژوهش از نوع مطالعات نیمه‌تجربی دوگروهی قبل و بعد بود که در آن 60 نفر از مراقبان اصلی سالمندان مبتلا به آلزایمر مراجعه‌کننده به انجمن آلزایمر ایران شرکت داشتند. این افراد به روش تصادفی ساده به دو گروه مداخله و کنترل تقسیم شدند؛ به‌صورتی‌که در هرگروه 30 نفر قرار گرفتند. ابتدا برای انجام پژوهش از دانشگاه علوم بهزیستی و توانبخشی مجوزهای لازم گرفته شد. سپس برنامه ذهن‌آگاهی به‌مدت شش جلسه هفتگی و هر جلسه به‌مدت دو ساعت برای گروه مداخله اجرا شد. به گروه شاهد هیچ برنامه مداخله‌ای ارائه نشد. داده‌ها از طریق پرسش‌نامه جمعیت‌شناختی و ابزار فشار مراقبتی رابینسون قبل و یک هفته بعداز پایان مداخله جمع‌آوری شد و با استفاده از نسخه نوزدهم نرم‌افزار SPSS و با آزمون‌های تی مستقل و تی زوجی در سطح معناداری (05/0) تحلیل شد. ملاحظات اخلاقی مطالعه حاضر عبارت بود از: محرمانه‌بودن اطلاعات افراد شرکت‌کننده، داوطلبانه‌بودن شرکت در مطالعه در تمام مراحل اجرای پژوهش و ارائه آموزش کامل برنامه ذهن‌آگاهی به گروه شاهد به‌دنبال مؤثر شناخته‌شدن مطالعه پس‌از انجام مداخله. یافته ها پس از انجام برنامه ذهن‌آگاهی، میانگین فشار مراقبتی در گروه مداخله از72/6±96/36 به 37/6±63/30 تقلیل یافت که نشان‌دهنده کاهش فشار مراقبتی در گروه مطالعه‌شده بود. این در حالی‌ بود که در گروه کنترل این میزان قبل و پس‌از انجام مداخله از61/7±56/35 به68/6±80/35 اختلاف معناداری نداشت. نتیجه گیری باتوجه‌به آنکه امکان کنترل همه متغیرهای پژوهش وجود نداشت، نتایج نشان می‌دهد مسئله فشار مراقبتی مراقبان سالمندان مبتلا به آلزایمر از مسائل بهداشتی مهم به‌شمار می‌آید که به توجه ارائه‌کنندگان خدمات بهداشتی نیاز دارد. همچنین راهکارهای جلوگیری و تسکین فشار مراقبتی این افراد باید در اولویت مراقبت‌های بهداشتی در جامعه مدنظر قرار گیرد. یافته‌های پژوهش نشان داد برنامه ذهن‌آگاهی موجب کاهش فشار مراقبتی مراقبان آلزایمر در این مطالعه شده است؛ ازاین‌رو کاربرد این روش به مراقبان پیشنهاد می‌شود.

Title: بررسی رابطه معنای زندگی و سلامت معنوی در سالمندان مقیم آسایشگاه‌های شهرستان شمیرانات، 1393

http://salmandj.uswr.ac.ir/article-1-1039-fa.html

اهداف سلامت معنوی از ابعاد مهم سلامتی به‌شمار می‌رود. اعتقاد به معنویت در تبیین هدف و معنای زندگی سالمندان نقش مهمی دارد. مطالعه حاضر باهدف بررسی رابطه معنای زندگی و سلامت معنوی در سالمندان مقیم آسایشگاه‌های شهرستان شمیرانات در سال 1393 انجام شد. مواد و روش ها پژوهش حاضر از نوع توصیفی تحلیلی بود که در آن 204 سالمند مقیم آسایشگاه‌های شهرستان شمیرانات در سال 1393 به روش نمونه‌گیری دردسترس انتخاب شدند. اطلاعات از طریق پرسش‌نامه سلامت معنوی پولوتزین و الیسون و پرسش‌نامه معنای زندگی (MLQ) جمع‌آوری شد. به‌منظور تجزیه‌وتحلیل داده‌ها از نرم‌افزار SPSS و آزمون ضریب همبستگی پیرسون و تی مستقل استفاده شد. یافته ها میانگین سلامت معنوی و معنای زندگی در سالمندان به‌ترتیب 65/21±96/72 و 3/93±03/53 بود. بین سلامت معنوی و معنای زندگی (31/0r= و 05/0P<)، بین سلامت وجودی و معنای زندگی (16/0r= و 05/0P<) و نیز بین سلامت مذهبی و معنای زندگی (15/0r= و 05/0P<) رابطه مثبت و معناداری وجود داشت. از میان متغیرهای جمعیت‌شناختی تنها سطح تحصیلات با معنای زندگی (12/0r= و 05/0P<) رابطه معنادار داشت. نتیجه گیری توصیه می‌شود عوامل همبسته و روش‌های مداخله‌ای مبتنی‌بر معنای زندگی در فرایند مراقبت از سالمندان مورد توجه و بررسی بیشتر قرار گیرد.

Title: شیوع سقوط و عوامل مرتبط با آن در سالمندان شهر بجنورد، 1394

http://salmandj.uswr.ac.ir/article-1-1117-fa.html

اهداف سقوط مشکل عمده بهداشتی است و صدمات ناشی از آن یکی از علل مهم مرگ و معلولیت در سالمندان محسوب می‌شود. هدف از این مطالعه بررسی فراوانی سقوط در سالمندان و عوامل مرتبط با آن در شهر بجنورد بود.  مواد و روش‌ها در سال 1394 این مطالعه مقطعی روی 288 سالمند 65 سال و بیشترِ ساکن شهر بجنورد و با استفاده از روش نمونه‌گیری طبقه‌ای تصادفی انجام شد. با همکاری مراکز مراقبت سالمندان، سالمندان بستری در بیمارستان و سالمندانی برای مطالعه انتخاب شدند که با خانواده زندگی می‌کردند. اطلاعات با استفاده از مصاحبه و چک‌لیست محقق‌ساخته‌ای جمع‌آوری شد که برای این مطالعه طراحی شده بود. متغیرهای این پژوهش علاوه‌بر اطلاعات جمعیت‌شناسی شامل این متغیرها بود: سابقه سقوط در شش ماه گذشته و متغیرهای مربوط به سلامت سالمندان و وضعیت بهداشت محیط محل زندگی آن‌ها. داده‌ها با استفاده از نسخه بیستم نرم‌افزار SPSS و آزمون‌های آماری تی مستقل و کای‌اسکوئر تحلیل شد. سطح معناداری 05/0≥ درنظرگرفته شد. یافته‌ها در این مطالعه شیوع سقوط در سالمندان شهر بجنورد 9/30درصد بود و 2/54درصد از نمونه‌ها را مردان و 8/45درصد را زنان با میانگین سنی 56/8±42/70 تشکیل می‌دادند. 75درصد از سالمندان بی‌سواد بودند. در زمان انجام مطالعه 1/10درصد سالمندان سیگار می‌کشیدند. میانگین تعداد نخ سیگار 20/3±03/7 در روز بود (کمترین2 نخ و بیشترین 10 نخ). میانگین مصرف دارو 98/1±44/2 قلم در روز بود (کمترین 1 قلم و بیشترین 8 قلم). 5/54درصد از سالمندان با خانواده و 8/27درصد در خانه سالمندان و 7/17درصد به‌تنهایی زندگی می‌کردند. ازنظر اشتغال 5/72درصد بیکار و 1/18درصد بازنشسته و 4/9درصد شاغل بودند. بیش از 50درصد سالمندان بیکار، از نهادهای حمایتی همچون کمیته امداد امام‌خمینی(ره) مستمری و خدمات دیگر دریافت می‌کردند. 1/55درصد از سقوط‌ها در داخل منزل یا مراکز مراقبت از سالمندان اتفاق افتاده بود. 4/92درصد سالمندان زیرپوشش بیمه‌های خدمات درمانی قرار داشتند. هیچ‌کدام از سالمندان مشروبات الکلی نمی‌نوشیدند. به‌ترتیب 4/49 و 5/22 و 1/28درصد سقوط بین ساعات 1:5 تا 12 صبح، 1:12 تا 18 عصر و 1:18 تا 5 شب و نیمه‌شب اتفاق افتاده است. همچنین 3/8درصد سابقه سکته قلبی، 9درصد سابقه سکته مغزی، 1/18درصد دیابت، 05/43درصد فشارخون و 9/4درصد کاهش وزن را گزارش کرده‌اند. در این مطالعه باتوجه‌به 05/0≥P بین وقوع حادثه سقوط و سن بالای 65 سال، مصرف داروهای خواب‌آور و آرام‌بخش، کمردرد، آرتروز، مشکلات راه‌رفتن، مشکلات نشستن و بلندشدن، مشکلات شنوایی و عوامل بهداشت محیطی همچون وضعیت پله‌ها و نرده‌های محل زندگی، وضعیت پوشش کف محل زندگی، وضعیت حمام محل زندگی، وضعیت سرویس بهداشتی محل زندگی و وضعیت روشنایی محل زندگی ارتباط معناداری وجود داشت. این در حالی است که بین سقوط و جنسیت، مصرف موادمخدر، تحصیلات، مصرف مشروبات الکلی، مشکلات بینایی رابطه معناداری مشاهده نشد (05/0 نتیجه‌گیری نتایج مطالعه نشان می‌دهد شیوع سقوط در سالمندان فراوان است. باتوجه‌به افزایش جمعیت سالمند در کشور و هزینه‌های زیاد درمانِ آنان، روش‌های مراقبت و پیشگیری باید بیشتر برای کاهش سقوط به‌کار برده شود. افزون‌براین کارشناسان مرتبط باید به‌طورمکرر ایمنی منزل را ارزیابی کنند.
